# Causes of Subcutaneous Inflammation and Suppuration

**Published:** 1887-08

**Authors:** P. Grawitz, W. De Bary

**Affiliations:** Virch. Arch. Fur Patholog. Anatomie in Physiology U. Fur Klinische Medicin. B’D. CVIII, 1887; Virch. Arch. Fur Patholog. Anatomie in Physiology U. Fur Klinische Medicin. B’D. CVIII, 1887


### ON THE CAUSES OF SUBCUTANEOUS INFLAMMATION AND SUPPURATION.

BY P. GRAWITZ AND W. DE BARY, (VIRCH. ARCH. FUR PATHOLOG. ANATOMIE IN PHYSIOLOGY U. FUR KLINISCHE MEDICIN. B'D. CVIII, 1887.)

[From a Review in the *Centralblatt fur Bacteriologie und Parasitenkunde, I., B'd.* No. 1, 1887, (Beumen, of Greifawald). Translated by Professor Frank S. Johnson.]

GRAWITZ, in the *Charité Annalen,* XI., 1886, under the title "Statestischer und Experimentell Pathologischer Beitrag zur Kenntniss der Peritonitis," stated his views upon the causal conditions of purulent inflammation of the peritoneum. He then expressed the opinion that the various bacteria of pus were not sufficient in themselves to cause purulent peritonitis, but that besides these specific causes of disease other forces inherent in the tissues must be brought into activity.

In the work at hand the same question is pursued in its relation to the subcutaneous tissues. Further investigations are contemplated to inquire into the causes of pleuritis. Grawitz and Bary consider the subject under four heads:

A. What is the effect of the absorption of unirritating fluids from the subcutaneous tissues.

The opinion held at the present time that the entrance of bacteria into the subcutaneous tissues, as the only exciting cause of suppuration, is not deserving of unqualified acceptance. It seems essential that the bacteria should form colonies and multiply in the tissues or upon wound surfaces before they are absorbed. When immediate absorption occurs they are harmlessly eliminated from the body.

B. What is the effect of irritating watery solutions?

(1). Concentrated salt and sugar solutions, either sterilized or containing staphylococcus aureus, injected into the tissues caused œdematous swelling, but no suppuration in dogs or rabbits. The addition of even considerable quantities of staphylococci was without effect. Suppuration resulted only when there was necrosis of the skin.

(2). The effect of acrid substances in watery solution.

I. *Group.* — Germ-destroying substances. Corrosive sublimate, 1 pe[illegible] cent.; nitrate of silver, 1 per cent.; abso lute alcohol, chloride of zinc, 1 per cent.

(*a*). Corrosive sublimate in dogs and rabbits underwent simple absorption,— in most instances it was accompanied by transitory swelling, but there was no suppuration.

(*b*). Experiments were conducted with nitrate of silver, both in sterilized solutions, and in solutions containing pus-microbes. The addition of the latter was without influence, inasmuch as the germ-potency of the microbes was destroyed by the solution (5 per cent., and much weaker). Very weak solutions (0.5 per cent.) were simply absorbed. Stronger solutions (5 per cent.) produced abscesses in dogs. The pus collected under aseptic conditions was free from germs.

It seems evident that suppuration is a reaction of animal tissues, which may be induced by a chemical substance without the influence of bacteria—a substance which may in fact destroy germs—provided the reagent is of the requisite strength, and is introduced into the tissues in sufficient quantity.

(*c*). The injection of absolute alcohol containing pus-microbes, or followed immediately by the injection of water holding the microbes in suspension, did not cause suppuration.

(*d*). Chloride of zinc in 1 to 5 per cent. solution gave the same results.

II. *Group.*—Soluble acrid substances, not germ-destroying. Acids, potassium and sodium hydrate and ammonia.

(*a*). The injection of acids, either in strong or weak mixture, did not produce suppuration. The addition of staphylococci (aureus, citreus, albus) did not alter the result.

(*b*). The hydrates of potassium and sodium gave similar results. The lye swelled the tissues and destroyed them. It did not produce favorable conditions for the growth of microbes within the body.

(*c*). Ammonia solution - sterilized. When introduced in small quantities and in weak solution it was absorbed without producing inflammation. Larger quantities (4-6 c. cm.), and stronger solutions caused abscesses in dogs. Cultures of this pus upon agar agar remained sterile. Repetition of this experiment, with the addition of pus-microbes, gave the same result, except that in the latter colonies of micrococci appeared on the agar agar. "These experiments show conclusively that ammonia of sufficient strength causes violent inflammation and suppuration. It provides suitable conditions in the subcutaneous tissues for the development of the microbes of pus. In very weak solution it does not injure the tissues.

C. How does the absorption of irritating oily substances act?

(*a*). *Turpentine.*—Preliminary experiments showed that turpentine was an active germicide. Its effect upon the subcutaneous tissues varied in different animal species. Its introduction in rabbits and guinea pigs, with or without the addition of pus-microbes, was followed by inflammation, but no suppuration. In dogs, on the contrary, it proved to be an active pus-producing agent.

(*b*). *Croton Oil* is not a germicide. Experiments showed that it was capable of exciting inflammation. In small quantities it caused in rabbits a watery or fibrinous exudation into the tissues. In larger quantities it corrodes; has a toxic effect, and, under certain conditions, may cause suppuration. When pus-microbes are added, suppuration always follows.

D. How does the injection of pus-microbes act?

Pus-microbes, in themselves, are not capable of inducing suppuration in healthy tissues—dogs and rabbits.

Various chemical substances can, without the aid of bacteria, induce suppuration under favorable conditions. When used in proper quantity, and in proper concentration, and upon animals whose tissues will react, suppuration invariably follows. Certain chemical substances in proper strength fit the tissues for the growth of pus-microbes.

Grawitz and Bary conclude that the same importance is attached to the ptomaines of pus-microbes as has recently been demonstrated for those of numerous other bacteria. The ptomaines of pus-microbes, acting as chemical irritants, prepare a soil in the subcutaneous tissue upon which the microbes may grow. Their effect is very readily seen upon the introduction of sterilized cultures, in which the fungus-poison is the only active agent. Sterilized cultures of the yellow pus-microbe induced suppuration in dogs. The pus contained no germs. For the formation of ptomaines the presence of free oxygen is of the utmost importance.

